# Leveraging artificial intelligence to advance implementation science: potential opportunities and cautions

**DOI:** 10.1186/s13012-024-01346-y

**Published:** 2024-02-21

**Authors:** Katy E. Trinkley, Ruopeng An, Anna M. Maw, Russell E. Glasgow, Ross C. Brownson

**Affiliations:** 1 Department of Family Medicine, School of Medicine, University of Colorado Anschutz Medical Campus, Aurora, CO USA; 2 Adult and Child Center for Outcomes Research and Delivery Science Center, University of Colorado Anschutz Medical Campus, Aurora, CO USA; 3 Department of Biomedical Informatics, School of Medicine, University of Colorado Anschutz Medical Campus, Aurora, CO USA; 4 Colorado Center for Personalized Medicine, School of Medicine, University of Colorado Anschutz Medical Campus, Aurora, CO USA; 5 Brown School and Division of Computational and Data Sciences at Washington University in St. Louis, St. Louis, MO USA; 6 School of Medicine, Division of Hospital Medicine, University of Colorado Anschutz Medical Campus, Aurora, CO USA; 7 Prevention Research Center, Brown School at Washington University in St. Louis, St. Louis, MO USA; 8 Department of Surgery, Division of Public Health Sciences, and Alvin J. Siteman Cancer Center, Washington University School of Medicine, Washington University in St. Louis, St. Louis, MO USA

**Keywords:** Implementation science, Artificial intelligence, Team science, Translational research, Learning health systems

## Abstract

**Background:**

The field of implementation science was developed to address the significant time delay between establishing an evidence-based practice and its widespread use. Although implementation science has contributed much toward bridging this gap, the evidence-to-practice chasm remains a challenge. There are some key aspects of implementation science in which advances are needed, including speed and assessing causality and mechanisms. The increasing availability of artificial intelligence applications offers opportunities to help address specific issues faced by the field of implementation science and expand its methods.

**Main text:**

This paper discusses the many ways artificial intelligence can address key challenges in applying implementation science methods while also considering potential pitfalls to the use of artificial intelligence. We answer the questions of “why” the field of implementation science should consider artificial intelligence, for “what” (the purpose and methods), and the “what” (consequences and challenges). We describe specific ways artificial intelligence can address implementation science challenges related to (1) speed, (2) sustainability, (3) equity, (4) generalizability, (5) assessing context and context-outcome relationships, and (6) assessing causality and mechanisms. Examples are provided from global health systems, public health, and precision health that illustrate both potential advantages and hazards of integrating artificial intelligence applications into implementation science methods. We conclude by providing recommendations and resources for implementation researchers and practitioners to leverage artificial intelligence in their work responsibly.

**Conclusions:**

Artificial intelligence holds promise to advance implementation science methods (“why”) and accelerate its goals of closing the evidence-to-practice gap (“purpose”). However, evaluation of artificial intelligence’s potential unintended consequences must be considered and proactively monitored. Given the technical nature of artificial intelligence applications as well as their potential impact on the field, transdisciplinary collaboration is needed and may suggest the need for a subset of implementation scientists cross-trained in both fields to ensure artificial intelligence is used optimally and ethically.

Contributions to the literature

- Artificial intelligence may be integrated into implementation science research and practice to enhance speed, sustainability, equity, and generalizability as well as the ability to assess context, context-outcome relationships, and causality. We highlight ways artificial intelligence can complement implementation science methods and provide examples.
- Using artificial intelligence with implementation science methods can also present new challenges and unintended consequences. We describe the potential pitfalls of using artificial intelligence, along with examples.
- We offer recommendations and resources on how to begin to responsibly integrate artificial intelligence into implementation science methods, including transdisciplinary collaboration and proactive monitoring for and mitigation of potential unintended consequences.

## Background

Healthy People 2030 vision calls for a “society in which all people can achieve their full potential for health and well-being” [1]. This vision is aspirational and leaves much to be done. Among high-income countries, the average number of annual deaths that could be avoided altogether with preventive or treatment strategies ranges from 130 to more than 330 per 100,000 people [2]. Although the lag time from knowledge generation to translation varies by situation, recent estimates suggest the average time is 15 years, which is a modest improvement from prior estimates [3, 4]. To move the needle and make real progress toward this vision, we need to do better faster, which includes producing and sustaining equitable results. We need more rapid knowledge generation and translation done in replicable, equitable, sustainable, locally relevant, and externally valid ways [5, 6]. Implementation science (IS) can play a key role in translating evidence into practice and policy.

IS methods and approaches can drive improvements in equity, sustainability, and the balance between local relevance and external validity needed to support translational science. When used by learning health systems or, more generally, in healthcare or public health settings, IS can iteratively support the continuum of knowledge generation to translation in many ways [7]. IS specifically focuses on feasibility and relevance to the local context while also considering principles of designing for dissemination, sustainability, and equity [8]. Importantly, equity is considered at each step in the continuum of knowledge generation to translation and promoted through the representation of partner perspectives and representativeness of outcomes [9–16]. However, IS has several limitations and challenges, notably the time and resources required to apply its methods and approaches. Such constraints can lead to reduced frequency, sample sizes, or representation of partner engagement and hamper other methods commonly used to assess context and outcomes [17]. Such limitations can dampen the potential for IS to enhance reach, equity, sustainability, and generalizability and ultimately impede its ability to close the evidence-to-practice gap.

As artificial intelligence (AI) gains prominence in the public health and healthcare sectors, it provides avenues to address some of the challenges to IS. AI algorithms such as machine learning (ML), deep learning (DL), and reinforcement learning (RL) serve as the foundation. Domain applications of these algorithms, such as natural language processing (NLP), are increasingly acknowledged as essential tools in the health sciences landscape [18]. See Table 1 for a description of key AI terms used in this paper. Their diverse applications range from predicting disease outbreaks, enhancing medical imaging, and refining patient communication via tools like chatbots to influencing behavior changes at patient, staff, organizational, or even community levels. Over the last decade, there has been a significant increase in the volume of scientific literature integrating AI into health research [19, 20]. This research incorporates a broad spectrum of AI models—from shallow ML algorithms, such as decision trees and k-means clustering, to deep neural networks. These AI models are applied to various data sources and types, such as clinical and observational data and data formats, including tabular, text, and images. The growth of large-scale, diverse health data, coupled with the emergence of new AI techniques, has led to significant change in the healthcare sector, improving our capabilities in diagnosis, disease prediction, patient care, and behavior modification [19–23]. AI technologies also afford opportunities to automate aspects of care delivery, quality improvement, and health services research processes that previously required human labor and thereby can increase speed and efficiency, including for implementation research and practice [24].

**Table 1:** Definitions of artificial intelligence terminology used

Artificial intelligence term	Definition
Artificial intelligence (AI)	An umbrella term for computer software that mimics human cognition to perform complex tasks and learn from them
Machine learning (ML)	A subfield of AI that uses algorithms trained on data to produce adaptable models that can perform a variety of complex tasks
Neural network	A machine learning technique designed to resemble the human brain’s structure. Neural networks require large data sets to perform calculations and create outputs, which enables features like speech and vision recognition
Shallow learning	Usually refers to the classic machine learning approach in the absence of neural networks
Deep learning	A subset of machine learning that uses several layers that form neural networks
Reinforcement learning (RL)	A type of machine learning in which an algorithm learns by interacting with its environment and is rewarded or penalized based on its actions
Natural language processing (NLP)	A type of AI that enables computers to understand spoken and written human language. NLP enables features like text and speech recognition on devices
Sentiment analysis	A systematic approach to understanding human language's affective and subjective intent, including whether data is positive, neutral, or negative
Decision trees	A type of machine learning that uses a decision tree to make predictions
k-means clustering	A type of machine learning which groups an unlabeled dataset into different clusters or categories

The potential of AI to enhance IS is evident, *but there are also cautions to consider*, including AI’s potential to exacerbate inequities if unchecked [25–28]. This paper aims to elucidate how AI can address current IS challenges while also shedding light on its potential pitfalls. We further provide specific examples from global health systems, public health, and precision health to illustrate both the advantages and precautions when integrating AI with IS. We conclude by providing recommendations and selected resources for implementation researchers and practitioners to leverage AI in their work. While there are extant primers on AI in healthcare and research and papers describing how IS can enhance AI [29–31], this paper focuses on ways AI can address challenges specific to IS and offers tangible guidance tailored to the IS community on how to apply AI to their work while being cognizant of and mitigating potential unintended consequences. We also discuss intellectual property rights related to the use of AI.

## Main text

### 2A. Opportunities for integration of AI to optimize IS methods

Here, we outline “why” AI should be used in the field of IS by describing some of the key challenges facing IS as well as tangible examples of how AI can help overcome these challenges. The specific IS challenges addressed are (1) speed, (2) sustainability, (3) equity, (4) generalizability, (5) assessing context and context-outcome relationships, and (6) assessing causality and mechanisms. Table 2 summarizes these IS challenges and AI solutions. Table 3 provides examples from health systems and public health settings describing how AI can address the limitations of IS.

**Table 2:** Implementation science challenges and artificial intelligence solutions with caveats

IS challenge (“why” use AI)	AI potential contributions	Potential adverse “consequences” of AI
Speed		
Qualitative partner engagement methods require personnel time to conduct	AI chatbots could be trained to moderate/lead	AI needs to be monitored to ensure prompts and questions are appropriate, responsive, and unbiased
Quantitative and qualitative data collection, analyses, and iterations require time	NLP/text mining can automate and speed up the process of: • Qualitative analyses • Collecting unstructured contextual data documented in electronic health records and other data sources AI algorithms can automate iterative data collection and analyses and display findings in accessible ways (e.g., dashboards) AI can replicate analyses and predictions with different subsamples when have sufficiently big data	AI needs to be monitored to ensure appropriate, complete, accurate, and replicable analyses, including for sentiment and inherent biases in data, as well as data drift
Intervention adoption is often slow due to difficulty testing implementation strategies that achieve behavior change and lead to adoption	With big data, AI can analyze some types of implementation strategies in almost real-time NLP and AI-enabled chatbots can create tailored messaging or nudges for clusters based on the characteristics of the intended adopters	Inherent ethical tensions with the use of nudges that should be carefully considered (e.g., ensure preserving autonomy and promoting positive change)
Sustainability		
Iterative evaluation of the dynamically changing context is important to improve sustainability but challenging to achieve given the time and effort required	Predictive AI algorithms can assist in assessing the chance of sustainment AI algorithms can continually work in the background to assess changes (e.g., dashboards) NLP can make ongoing/iterative qualitative assessment more efficient and feasible	AI needs to be monitored for data drift and biases in data AI needs to be monitored to ensure appropriate, complete, and accurate analyses, including for sentiment
Equity		
Language, digital literacy, and social needs can be barriers to participating in: • Partner engagement • Implementation studies • Intervention education implementation strategies	AI-enhanced translation software (i.e., Google Translate) can increase access to and inclusion	Patients with these characteristics are often less likely to be part of AI data sets- (e.g., genomic registries, electronic health record databases) Messages through translation software may be distorted, mistrusted, or insensitive/off-putting
People from historically marginalized groups may not respond to risk communication, behavior change, or decision-making messaging that is effective in other groups or experience mistrust	Culturally tailored AI-enabled messaging or chatbots can support the adoption of EBI in historically marginalized groups AI-enabled chatbots may be able to improve racial empathy gaps and provide feedback on issues such as social determinants, and risk status [32]	Use of nudges could have ethical implications if not monitored or used responsibly Could alienate groups further if not done well or relying on superficial “surface” disparity information Could perpetuate distrust of the medical system and lead to distrust regarding the purpose of data collection Inaccurate predictions could result from biased or missing data
Historically marginalized groups may experience large disparities and are often under-represented in surveillance systems, surveys, and other data sources	AI can expand data sources and recruit participants through various online and other channels to increase the breadth of representativeness	Could worsen the underrepresentation of certain groups and study conclusions if not carefully considered
Generalizability		
Scope/reach of partner engagement may be limited or may not reach “right” people due to time and manpower required to conduct engagement methods	AI can recruit participants through various online channels, improving the breadth of perspectives and data quality while greatly reducing the human power required AI chatbots could be trained to moderate/lead or generate agendas and recruitment materials (e.g., ChatGPT)	Need to monitor for new biases, inequities or unintended consequences introduced or created due to: • irrelevant or partial samples of populations • analyses of biased data AI needs to be monitored to ensure prompts and questions are appropriate, responsive, and unbiased
Surveys and other traditional D&I data sources may be limited to partial or small samples and represent limited partner perspectives	AI can utilize social media and other secondary data sources, including observational data to glean valuable insights about population trends and public sentiment AI can generate synthetic datasets that closely mimic real-world data sources. These synthetic datasets, created based on patterns and structures found in real data, can supplement existing survey data, thereby broadening the scope of analysis without incurring additional data collection costs AI can deploy surveys effectively through various online channels, potentially improving response rates and data quality while reducing the human power required AI can assess sustainment of generalizability over time with continued vigilance post-intervention	Without monitoring new biases, may lead to inequities or unintended consequences introduced or created due to: • inaccurate sentiment analyses • incomplete population samples • inclusion of biased data • generation of biased data sets May lead to a lack of transparency when developing synthetic datasets and considering regulations Data may not be available for AI to assess for key issues, such as trust or racism
Assessing context and context-outcome relationships		
Traditional approaches to understanding context/determinants is limited to the “stated” (e.g., qualitative partner engagement, surveys) and the “realized” (e.g., quantitative data sources) that require an a priori signal and are usually cross-sectional evaluations	AI algorithms can digest large amounts of quantitative data, including from non-traditional data sources (e.g., wearables), and look for patterns in contextual variables and de novo signals, allowing for the identification of issues/problems/inequities that we didn’t know to look for Data sources could include observations of activities or processes (e.g., time stamps or clicks within the electronic health record) to extend contextual data to assess Can replicate AI algorithms to look for stability over time or in different settings or contextual situations	Need to consider implications of “black box” algorithms and whether “explainability” and validation or further exploration is needed—consider regulations and recommendations
Measures of organizational culture are important for implementation but are underdeveloped and seldom available on large and representative samples	NLP with sentiment analyses can be applied to data sources such as emails, text messages, and videos to measure culture	Monitoring is invasive to members of the organization and presents new ethical considerations
Assessing causality and mechanisms		
Quantitative analyses in D&I are often descriptive, are rarely comparative, and do not account for complex and non-linear interactions between context/determinants or outcomes, which limits causal assessments of outcomes under specific contextual situations for different: • IS TMFs used • Interventions • Implementation strategies	AI algorithms can account for the intangible interactions between data, leading to new insights AI algorithms can compare outcomes of exposure groups using complex and large data sets When examining the multilevel context in a D&I study, AI may allow us to examine interactions and effects across levels	Need to consider implications of “black box” algorithms and whether “explainability” and validation or further exploration is needed—consider regulations and recommendations

*AI* artificial intelligence, *IS* implementation science, *NLP* natural language processing, *EBI* evidence based intervention, *D&I* dissemination and implementation, *TMFs* theories, models, and frameworks

**Table 3:** Examples of how AI can address IS challenges in health systems and public health settings

IS challenge	AI example
Speed	NLP was used for qualitative analysis coding and compared to a traditional, non-AI-enhanced approach. The authors found that NLP can identify major themes, but that traditional approaches were best at identifying more nuanced details [33]
	AI-enhanced chatbots were designed to identify and address barriers to chronic medication adherence with messages tailored to patient characteristics and needs [34]
Sustainability	Case study showed how AI can improve data analysis and improve the efficiency of clinical processes and, thereby, improve sustainability [35]
Equity	AI-enhanced chatbots were created to provide culturally relevant education and support for new mothers and to address health disparities [36]
	Demonstrates how AI can be used to identify ongoing clinical trials that historically underrepresented patients are eligible for [37]
Generalizability	AI was applied to social media data to identify adverse events and public sentiment associated with immunizations in a large and heterogeneous population [38]
Assessing context and context-outcome relationships	AI was used to identify contextual reasons for clinician non-adherence to guideline-recommended practices, including identification of previously unrecognized issues [38]
	AI was used to predict coronary artery disease and quantify death risk using electronic health records and genetic data [38]
Assessing causality and mechanisms	Describes how AI was used to create actionable and individualized causal treatment effect predictions for patients with Alzheimer’s disease [39]

*AI* = artificial intelligence, *IS* = implementation science, *NLP* = natural language processing

### 2A1. Speed as an IS challenge

Improving and measuring the speed of its methods and translation is a critical issue for IS [17, 40, 41]. Despite great promise and evolving methods to improve the speed of certain activities, IS methods require time, including time to conduct partner engagement, test implementation strategies, evaluate outcomes, and collect and analyze mixed methods data. The time required to carry out traditional IS approaches can slow the speed of knowledge generation and translation, which can be expedited with AI. For example, AI-enabled chatbots can be trained to lead or moderate qualitative interviews or focus groups, allowing for multiple sessions to be completed in parallel without the usual constraints of personnel available. There are examples of such chatbots that are already being used in the business sector for job candidate interviews [42], which could be adapted for IS applications. NLP and more advanced forms of AI can also be used to collect and analyze data inductively or deductively, including the collection of unstructured data that typically requires manual analysis of qualitative data that is traditionally time-consuming and slow [43]. The use of AI to conduct qualitative analyses is becoming more common, either as a standalone method or in a “human-assisted” method where researchers iteratively review the AI outputs and provide redirection as needed [44–47]. Newer, rapid approaches to qualitative analysis in IS have already sped up this step [48], but these newer analysis methods could also be augmented with AI to expedite or supplant person time by an order of magnitude. Notably, AI-enabled software is readily available to assist with transcription [49]. Table 3 summarizes a study that compared NLP to traditional qualitative methods and found NLP was effective at identifying major themes but was not as precise at more granular interpretations [33]. Chatbots can also be used to automate the creation and testing of tailored messaging as educational implementation strategies (e.g., behavioral nudges) for different target groups of patients, staff, or settings based on their unique characteristics to increase the speed of identifying contextually appropriate and effective strategies [50]. Most examples of leveraging AI to accelerate speed are currently outside the field of IS [42, 44–47].

### 2A2. Sustainability as an IS challenge

Sustainability is a central tenant of IS and ideally requires iterative, ongoing progress assessments to identify intervention components and implementation strategies needing adaptation [51–54]. However, these ongoing evaluation methods can tax available resources, particularly human capital. By automating iterative evaluation cycles, AI may reduce the demand for human resources, which is often a bottleneck to sustainability methods. A study conducted by the Regional Social Health Agency in Italy (Table 3) demonstrates how AI can improve the efficiency of using health information and promote the sustainability of healthcare systems [35]. There are other avenues in which AI can contribute to sustainability. For instance, AI algorithms can be configured to continuously monitor for and work in tandem with chatbots and NLP tools to detect subtle changes in outcomes that are difficult for traditional quantitative approaches to detect within complex and big data sets used within healthcare. These algorithms can provide partners with real-time insights through integration with platforms like dashboards [55–57]. To date, there are examples of dashboards being used to make such sustainability methods feasible for IS projects [58], but there are few examples of AI-enabled approaches in the field of IS. Such use of AI with dashboards can be particularly useful when rapid decisions are required or when partners need to identify and understand complex or subtle patterns in data over time.

AI’s predictive analytics could also be employed to simulate or forecast the sustainability of certain initiatives and estimate the long-term viability of a project or implementation strategy [59]. For example, if an intervention is implemented in a healthcare setting, AI could analyze data on adherence rates, participant feedback, and other relevant metrics to project the likelihood of its continued success. IS frameworks could guide the systematic assessment of the complex and multilevel contextual factors (e.g., culture, strategic priorities, burnout rates, turnover) that influence sustainability which could be categorized into themes and used as input or predictor variables within the AI model. Such predictive capabilities could allow for proactive and iterative adjustments throughout the life of a project to maximize sustainability. This approach could also assist in identifying the optimal allocation of limited resources by knowing in advance which areas might falter or by potentially supplanting the need for a costly or time-consuming trial that is predicted to be unsustainable. The use of AI to predict sustainability is a potential future direction for the field of IS.

### 2A3. Equity as an IS challenge

IS aims to promote equity, but some equity-enhancing activities can be challenging within resource constraints. Resources are often not available to (1) disband language barriers to participation in partner engagement activities and implementation studies; (2) create culturally appropriate implementation strategies that address issues such as mistrust; or (3) offer data that represent the spectrum of perspectives beyond the usual, including that of persons who have historically been marginalized and experienced disparities [60, 61].

IS can benefit from integrating AI to promote equity amidst resource constraints. AI-driven translation tools render text in different languages and can capture the essence and nuances across dialects and regional variations. Further, speech-to-text systems can convert spoken language into written form, facilitating participation for those who might be literate in their native tongue but not in the primary language of a study. For immediate interactions, real-time AI-enhanced software interpretation allows non-native speakers to understand and contribute actively. AI chatbots, tailored using user data and historical contexts, can resonate with local customs and beliefs, offering a culturally attuned interaction [62, 63]. Additionally, these AI systems can be trained to transparently provide resources that resonate with targeted communities and can be employed for cultural awareness training, ensuring researchers approach communities with heightened sensitivity [62, 63]. In terms of data, AI algorithms can increase diverse representation by pulling from a range of sources, inclusive of historically marginalized voices that are often omitted from traditional datasets because the data are in unstructured formats and/or too large and complex for traditional analytic methods [9].

In Table 3, we present an example of proactively using AI to identify clinical trials for patients from historically underrepresented populations [37]. These AI tools can also be configured to detect and rectify inherent biases in datasets and present complex data visually, aiding in identifying and correcting disparities. For recruitment, AI’s ability to analyze complex population data from diverse sources means that underrepresented groups can be pinpointed for more inclusive outreach [64]. Data sources could include social media platforms, online community forums, or leverage crowdsourcing techniques. AI-driven tools such as voice assistants and adaptive interfaces could also be used to make research platforms more navigable for those with disabilities or language barriers [65]. Finally, AI’s feedback mechanisms enable real-time adjustments to implementation strategies based on participant input, and sentiment analysis tools can gauge the emotional underpinnings of this feedback, illuminating areas of potential mistrust or dissatisfaction [66]. In harnessing these AI capabilities, IS can promote equity more effectively, ensuring historically marginalized communities are actively engaged in research and its applications. The use of AI to promote equity remains largely untapped, with most examples outside the field of IS [60, 61, 64–66].

### 2A4. Generalizability as an IS challenge

Although IS prioritizes generalizability and transportability [67], limitations of data and human resources to conduct partner and participant engagement and collect data can threaten generalizability. Generalizability decreases if the breadth of perspectives considered is limited when designing, implementing, or evaluating a study [68]. While AI’s role in easing resource demands through chatbots and NLP has been acknowledged, AI’s potential to enhance generalizability stretches beyond that. Because AI can sift through large amounts of complex data, it can incorporate insights from non-traditional sources. For example, social media platforms, with user-generated content, can provide rich insights into public sentiment, behavior, and preferences, which increases the representation of perspectives and assists in generalizing findings across diverse populations [69]. The United Kingdom has applied AI to Twitter and Facebook forums to evaluate adverse reactions and understand public sentiment toward the COVID-19 vaccination and found that common and rare adverse effects were discussed with relatively equal frequency and that vaccine perceptions were largely positive over time (Table 3) [70]. Additionally, AI can use crowdsourcing to increase the representation of diverse perspectives [71, 72]. Crowdsourcing has the potential to capture diverse insights from global audiences. AI has been used to coordinate and process data from large, crowdsourced projects, ensuring that perspectives are drawn from a cross-section of diverse individuals [73]. This means that studies can encompass views from varied geographical locations, socio-economic statuses, and cultural backgrounds while operating within existing resource confines [74]. As is also the case with traditional data sources, the selection of an appropriate social media or crowdsourcing data source must be aligned with the target population or issue at hand to ensure relevance, and inherent data biases, including misinformation and missingness must be considered.

Moreover, the dynamic nature of the world means that generalizability is not static. Populations evolve, cultures shift, and societal priorities change. Here, AI can assist by automating continuous assessments of generalizability. Similar to approaches described above related to sustainability, AI can monitor for changes that might impact the external relevance or transportability of study findings and provide alerts or updates when shifts are detected. Such iterative assessments, automated by AI, can be used to strategically guide adaptations such that the work and findings remain generalizable throughout all stages of a study and over time [57, 60, 61].

### 2A5. Assessing context and context-outcome relationships as an IS challenge

Traditional IS approaches to assessing context and outcomes are often limited to the “stated” or simple interpretations of the “realized.” While the stated (explicit declarations) often come from qualitative methods like partner engagement sessions or surveys with limited samples, the realized is generally garnered from quantitative data necessitating a predefined hypothesis or signal [75]. Although emergent configurational analysis techniques delve deeper into intricate relationships between context and outcomes [76], IS and traditional quantitative approaches often fall short of capturing the intricate relationships of non-linear interactions. AI algorithms present new opportunities to address these challenges that are often inherent in complex data. AI can assimilate large and complex data repositories to discern non-linear relationships and detect patterns or context—implementation strategy—outcome relationships even without predefined signals [77–79]. One study leveraged AI and electronic health record data to understand reasons for gaps in clinician prescribing for a clinical scenario that had already been well studied using traditional mixed methods [75]. This study identified a variety of contextual determinants, including some that were previously unrecognized and were used to inform the design of an ongoing IS trial (Table 3) [75]. The versatility of AI means that these algorithms are not only static tools, but that they can be optimized to constantly operate in the background, evolving with the data they encounter. This becomes particularly crucial in dynamic landscapes such as healthcare, where relationships between context and outcomes can change rapidly. As AI iteratively processes this information, it can provide a pulse on any emerging shifts, ensuring that IS remains responsive and adaptive to the changing context. While there are limited examples of AI being used to assess context for IS studies [75], there are additional avenues in which to explore how AI can be leveraged to assess changes in context, strategies, and outcomes.

### 2A6. Assessing causality and mechanism as an IS challenge

In IS, ascertaining causality and mechanism is difficult. While traditional quantitative tests for causality could assist [80, 81], as could more qualitative approaches such as mechanism mapping [82], deciphering the direct causal connections, rather than mere associative links, between interventions, implementation strategies, and outcomes, is difficult due to the complex interplay of confounding variables in real-world settings. Modern AI-driven causal inference and discovery mechanisms offer a path forward for IS [83–85]. Leveraging structured graphical models, techniques such as causal Bayesian networks adeptly delineate explicit cause-and-effect relationships, duly accounting for latent confounders. Consider, for example, a healthcare scenario aimed at curtailing hospital readmissions. Whereas traditional analytical frameworks might predominantly identify an associative link between an intervention and reduced readmissions, AI causal tools probe deeper, scrutinizing whether the intervention itself was the direct catalyst or if obscured variables intervened. In Table 3, we provide a precision health example of using causal AI methods to generate treatment predictions for patients with dementia [39]. Further enriching the AI toolkit are counterfactual neural networks [86, 87], which could be used by IS practitioners to simulate hypothetical outcomes that would have occurred without specific interventions. Another notable advancement is AI's deployment in analyzing potential or simulated outcomes, which elucidates the individual treatment effect, thereby shedding light on the distinct impact of interventions or implementation strategies on specific demographic or clinical subgroups. Such AI-based simulation models have the potential to save unnecessary resource expenditure (time, money) if a trial is predicted to produce a null effect. Through this AI-driven lens, IS could better assess causality and mechanism with heightened precision, fostering the design and deployment of increasingly effective and equitable programs. The use of AI to assess causality and mechanism is a largely unexplored methodologic area for the field of IS.

### 2B. Potential consequences of using AI in IS

The consequences of using AI can be both positive and negative. Thus far we have focused on those positive consequences that can be anticipated, but there are likely others that are unanticipated. For example, it is not yet known what the true potential of AI is, and AI-generated innovations could create solutions with benefits we cannot begin to predict. However, when using AI, important considerations and potential adverse unintended consequences need to be monitored and minimized [25–28]. Here we highlight potential cautions of using AI with examples of how AI has caused harm or gone awry. In Table 2, we explicitly relate these AI concerns with the AI solutions proposed above to address IS challenges. Across all of these counterarguments, *proactive vigilance is required to identify and mitigate issues at each stage of the AI lifecycle*, which includes (1) data creation, (2) data acquisition, (3) model development, (4) model evaluation, and (5) model deployment [88]. We provide examples showing how AI can lead to erroneous conclusions, inequities, biases, or harmful behaviors.

Unmonitored AI applications (e.g., AI algorithms, chatbots, NLP) can lead to erroneous messages or results. AI is restricted to the available data inputs and subject to all the biases of the data collection process, often referred to as “garbage in, garbage out.” For example, it is known that clinician diagnoses are biased by gender and race [25, 26], and models using such data will capture these biases. AI’s sentiment analysis can also incorrectly interpret data or be influenced more by counts or frequencies than a manual human-only process would be, and such errors can significantly influence results [43]. These issues can be hidden or exacerbated when using “black box” AI models that result in an effect or outcome but do not allow for explainability of the processes that produced the effect [89].

In one study, an AI algorithm was applied to 14,199 patients with pneumonia across 78 hospitals to risk-stratify the probability of death [90]. The model recommended that patients with asthma were at lower risk than those without asthma. This recommendation contrasted with existing evidence, thus triggering the researchers to investigate further. The researchers discovered that the data inputs biased this finding. Specifically, the data inputs did not capture the fact that patients with asthma and pneumonia were commonly directly admitted for treatment and thus had better treatment outcomes compared to patients who had pneumonia without asthma.

AI also has the potential to exacerbate or create new inequities. Reliance on data that underrepresent the population or that are subject to inherent biases stemming from sexism, racism, classism, or mistrust leads to inaccurate predictions or evaluations and could perpetuate inequities or misguide decision-making [26–28]. Misguided decision-making can be particularly apparent when AI is used to inform recommendations for tools such as clinical decision support within electronic health records.

Authors of another paper provide a use case of AI algorithms scheduling medical appointments to improve scheduling efficiency to illustrate how such AI algorithms can yield racially biased outcomes [91]. Such algorithms consider many factors, such as characteristics of patients that arrive late to appointments or “no show.” However, historically, Black patients have a higher likelihood of “no shows”; thus, the algorithm scheduled these patients into less desirable appointment times.

AI is beholden to the data inputs. Beyond inherent biases of how data are collected, data inputs are also subject to data drift (e.g., temporal changes in how and where data is documented) and can also lead to biased interpretations if the sample sizes are not representative or sufficiently large [92]. Traditional IS data sources have limited sample sizes, and data drift is common in rapidly changing environments where public health and healthcare happen.

In 2009 it was announced that by using AI applications and publicly available data from Google search engine queries for “flu-like symptoms,” researchers could predict regional flu trends 1-2 weeks earlier than the Centers for Disease Control and Prevention [93]. Later, it was discovered that the prediction was no longer accurate, in part because of changes to search engines that prompted or suggested certain search terms to users, which changed the data inputs [94].

AI can tailor messages or nudges for specific populations in ways that prompt and facilitate good decision-making [95, 96]. However, such AI applications can also inadvertently promote harmful behaviors. For example, AI has been leveraged to create tailored messages or nudges to increase consumer uptake of unhealthy food and beverages [97]. AI can learn and adapt its messaging over time, thus posing the potential for messages originally well-intended to encourage inadvertent harm. Other ethical implications of nudges include situations in which certain options are forbidden and autonomy in decision-making is impaired [98–100].

### 2c. Intellectual property rights of using AI for IS

Intellectual property issues present unique challenges and opportunities when AI is used in IS for design, data analysis, or reporting [101]. Questions arise about property rights of the knowledge generated from AI models, especially when the knowledge generated stems from data in which it is unclear who owns the data. For example, AI models could leverage data sourced from public datasets or collaborative efforts in which ownership of the data is unclear. Furthermore, as AI aids in creating or optimizing interventions, discerning the boundaries between human-generated property and machine-augmented contributions can become ambiguous. It is imperative for researchers and practitioners to proactively navigate these complexities, ensuring that while AI propels IS forward, it does so in a manner that respects and delineates intellectual property rights and contributions.

If relying on existing AI applications, it is important to identify and understand any potential intellectual property rights, which could require fees for use or restrictions on how the AI can be used or disseminated. On the flip side, if creating de novo AI applications, it may be prudent to consider establishing intellectual property rights to enforce responsible use and avoid the potential consequences outlined above. Intellectual property rights apply to any invention, such as EHR-based tools and decision aids, but in the case of AI, intentional use to promote responsible AI use may be novel and important to consider. While there are clear implications of intellectual property rights for AI applications themselves, there is less clarity regarding the property rights of AI-generated products [102, 103]. The latter is a new and developing area that is currently handled on a country-by-country basis. Although allowable under the laws of some countries such as the UK, the US stance is that AI-generated products are prohibited from intellectual property rights [103]. The fundamental question that served as the basis for the US’s decision was “How can a thing (not a human) own property?” The inability to predict or anticipate AI-generated products confounded by limited means of regulation is cause for increased caution and monitoring.

## Discussion and future directions

IS and AI can complement each other and have the potential to work together to increase the speed of sustainable and equitable knowledge generation and translation to enhance healthcare and population health. We have focused on how AI can augment specific bottlenecks faced by the field of IS, while others have called attention to ways in which IS can augment AI, which includes making AI more relevant to local settings, scalable, and sustainable [29–31]. In summary, key ways AI can help address IS-specific challenges include: increasing the speed with which data can be collected, analyzed, and acted on; automating and reducing the workforce required to conduct partner engagement and other IS methods; expanding the size and heterogeneity of available data sources and participant recruitment; and increasing access to new methods to assist in discovering contextual influences and complex interactions between context, implementation strategies and outcomes. Focusing solely on AI’s potential to automate many of IS’s traditional methods and processes, AI provides a path to help IS researchers and practitioners become more rapid and achieve goals of sustainability, equity, and generalizability.

AI presents new opportunities for IS and many potential AI applications remain largely unexplored or untapped by the IS community. As AI use assuredly increases, it needs to be monitored and used responsibly to avoid unintended consequences, especially in the face of limited regulations on AI. It is also important to note that the benefits and pitfalls of AI may not equally apply to all types of AI and it is beyond the scope of this paper to address each separately, but the reader should keep in mind that there are differences based on the specific AI model and application. Among AI’s potential to cause harm, inequities have received much attention and may be one of the most challenging issues to monitor and mitigate. Inequities can surface over time, and multiple root causes include biased data inputs or data that do not represent the spectrum of cultures or perspectives. In this paper, we describe ways AI can optimize equity, but every use of AI also requires careful and ongoing vigilance for potential effects on inequities. Other potential pitfalls of AI discussed above include inaccurate predictions, recommendations, or interpretations of data. Another key challenge of AI is its algorithms’ reproducibility or “brittleness” across settings and over time [104]. There is a need for the regulations, frameworks, and guidance currently being developed for AI [105, 106] to include policies and procedures for systematic and proactive monitoring of unintended consequences and careful consideration of “black box” models [89]. With the widespread update and examination of ChatGPT (and other AI online and related tools), there is increasing awareness about AI’s potential for errors [107–109]. The full potential of using AI to enhance IS is not yet known, nor is its potential for errors and harm, which makes the development of regulations even more challenging [101].

IS should take full advantage of AI’s benefits while being mindful of its pitfalls. To do so, a transdisciplinary team science approach is optimal. Team science certainly extends beyond AI and IS partnering, but we focus on these two fields here. Historically, the fields of AI and IS have had limited collaboration with different foci (e.g., heavily quantitative and causal versus mixed methods and pragmatic effectiveness). Now as each becomes essential to the vision of precision public health and learning health systems [7, 110–115], they are progressively realizing the value of each other. Given both are rapidly evolving fields and that it is hard to anticipate what is new or next, close collaboration or perhaps a new generation of cross-trained scientists is needed. Such cross-trained scientists may be particularly adept at keeping pace with the latest discoveries related to AI’s potential and monitoring for and mitigating unanticipated consequences. To foster this budding partnership or cross-training between IS and AI, accessibility of expertise and resources is important. In Table 4, we provide a select sample of resources and tools to facilitate the use of AI especially relevant for IS.

**Table 4:** Select resources and key references especially relevant for IS to learn about artificial intelligence

Resource	Description (and costs)
RapidMiner https://rapidminer.com	A data science platform that aims to simplify AI for users, including those without coding experience *Requires paid subscription, but may be offered through institutional licenses*
Tableau https://www.tableau.com	A data visualization platform that includes native AI software that can be applied to data stored within *Requires paid subscription, but may be offered through institutional licenses*
ChatGPT https://chat.openai.com	An AI-driven chatbot that responds to user prompts. Examples of responses to prompts can include drafts of medical necessity letters, manuscripts, and data visualizations (graphs, tables). There are different versions of ChatGPT and similar tools of varying sophistication and accuracy *Free versions are available*
Example Data Science Training Programs: https://ischoolonline.berkeley.edu/data-science/ https://brownschool.wustl.edu/academics/aibda-certificate/ https://brownschool.wustl.edu/resources-initiatives/advanced-learning-certificate/artificial-intelligence-applications-for-health-data/ https://catalog.ucdenver.edu/cu-anschutz/schools-colleges-programs/graduate-school/graduate-school-certificates/biomedical-data-science-certificate/	Many academic universities offer graduate degrees, certificates, or boot camp-type programs in data science and other areas that provide AI training for diverse audiences *There is a cost*
Coursera https://www.coursera.org/collections/best-machine-learning-ai	Offers many online courses and programs in data science, AI, and different focus areas of AI *There is a cost but may be offered through institutional licenses*
You Look Like a Thing and I Love You: How Artificial Intelligence Works and Why It’s Making the World a Weirder Place[116]	A lighthearted easy-to-read book for those with some or no baseline knowledge of AI. The author uses practical examples to illustrate how AI works and its limitations without going into technical details or jargon *There is a cost to purchasing the audio or text*
Journal articles providing guidance on NLP for qualitative analysis: Chang T, DeJonckheere M, Vydiswaran VGV, et al.. Accelerating mixed methods research with natural language processing of big text data. J Mix Methods Res 2021;15:398–412 [117] Lennon RP, Fraleigh R, Van Scoy LJ, Keshaviah A, Hu XC, Snyder BL, Miller EL, Calo WA, Zgierska AE, Griffin C. Developing and testing an automated qualitative assistant (AQUA) to support qualitative analysis. Fam Med Community Health. 2021 Nov;9(Suppl 1):e001287 [118]	Illustrative examples and recommendations for how NLP can be used for rapid qualitative analysis *There is a cost to some of the papers, but some may be free through institutional licenses or open access*

*AI* artificial intelligence

## Conclusions

We call for increased uptake of innovations in AI through transdisciplinary collaboration to overcome challenges to IS methods and to enhance public health and healthcare while remaining vigilant of potential unintended consequences. We acknowledge that our paper is “first generation” in that it is one of the first to describe intersections between AI and IS—in doing so, we hope to spark future debate, scholarship, and enhancement of the concepts we have introduced. In Table 5, we provide concrete summary suggestions of how to begin to responsibly and optimally use AI, including building a representative team. Application of AI is complex and uncertain, but has the potential to make IS more efficient and can facilitate more in-depth and iterative contextual assessment, which in turn can lead to more rapid, sustainable, equitable, and generalizable generation and translation of knowledge into real-world settings.

**Table 5:** Recommendations to responsibly and optimally use AI in implementation research

Build effective transdisciplinary teams	That includes AI and IS expertise and representation of other perspectives, including other expertise, patients, community members, caregivers, staff
Have a creative mindset	AI presents a new opportunity to innovate. Think and act creatively while being cautious of pitfalls
Be audacious, humble, and persistent	Explore the potential of AI while anticipating things might go wrong and planning for iterations to check your work and correct course
Insist on and monitor equity	Evaluate and advocate for the representation of perspectives and datasets as well as representativeness of outcomes
“Fail early, fail often, but always fail forward”^a^	Be iterative to improve, including sustainability and equity
Conduct rapid learning	AI and IS are changing rapidly. Establish a process for staying up-to-date on the latest developments affecting their integration

*AI* artificial intelligence, *IS* implementation science

^a^Quotation attributed to John C. Maxwell

## Data Availability

Data sharing is not applicable to this article as no datasets were generated or analyzed.

## Abbreviations

AI: Artificial intelligence
DL: Deep learning
D&I: Dissemination and implementation
EBI: Evidence-based intervention
IS: Implementation science
ML: Machine learning
NLP: Natural language processing
RL: Reinforcement learning
TMFs: Theories, models, and frameworks
